# Indocyanine green fluorescence detects the blood flow of the bronchial anastomosis for bronchoplasty “case report”

**DOI:** 10.1016/j.amsu.2020.09.034

**Published:** 2020-09-28

**Authors:** Hidetaka Uramoto, Nozomu Motono

**Affiliations:** Department of Thoracic Surgery, Kanazawa Medical University, Ishikawa, Japan

**Keywords:** Indocyanine green fluorescence, Bronchial anastomosis, Bronchoplasty

## Abstract

**Introduction:**

Bronchoplasty is widely accepted as a standard technique with a high degree of difficulty in maintaining a surgical margin for non-small-cell lung cancer (NSCLC). The key to the success of the bronchial anastomosis is both tension and the blood flow. However, local tension is inconsistent with blood distribution.

**Case presentation:**

Operative finding of the right upper bronchoplasty after chemoradiotherapyshowed clear green staining of the upper bronchus, and afterwards, a membranous area of the truncus intermedius. The blood supply of the bronchial anastomosis judged to be enough.

**Discussion:**

Indocyanine green imaging (ICG) can help a scheduled operation be performed safely, especially in extreme situations where there is concern about the blood supply during bronchoplasty.

**Conclusion:**

This report describes a first case concerning the blood distribution of the bronchial anastomosis for bronchoplasty after induction therapy under fluorescence navigation.

## Highlights

• Our intraoperative findings clearly showed both the green staining of the upper bronchus and later a membranous area of the truncus intermedius.
• Indocyanine green fluorescence detects the blood flow of the bronchial anastomosis for bronchoplasty.
• These marvelous findings lead a planned operation safely in the extreme, especially in extreme situations where there is concern about the blood supply during bronchoplasty.

## 1 Introduction

Lung cancer is the leading cause of death worldwide, and bronchoplasty are occasionally necessary to avoid pneumonectomy and spare lung functions for central-type NSCLC [1]. The key to the success of the bronchoplasty is both tension and the blood flow. However, these two factors has a relation between contraries. We experienced a case of clear green staining of the upper bronchus, and afterwards, a membranous areas of the truncus intermedius under fluorescence navigation.

## 2 Case Presentation

**Fig. 1:**
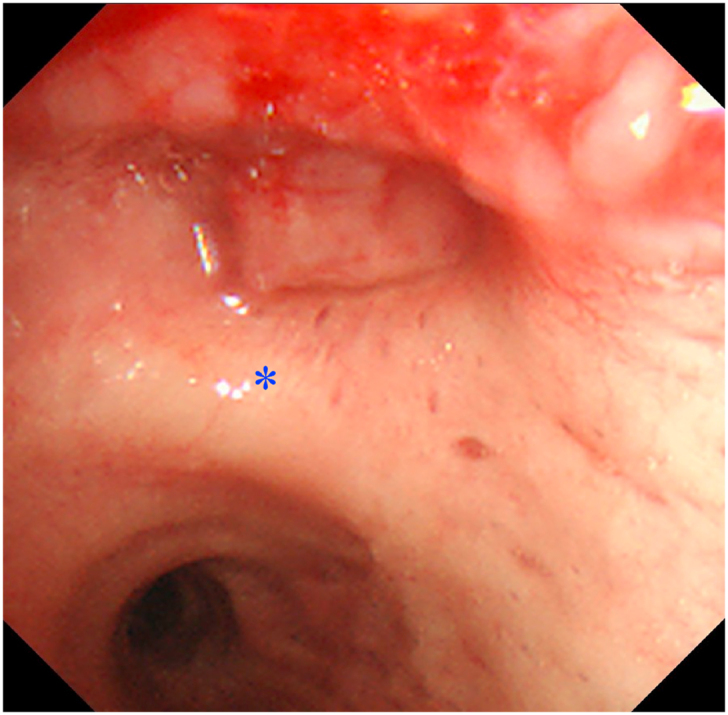
Bronchoscopy shows an obtuse second spur of the right bronchus and obstruction of upper bronchus. *: bifurcation between right upper bronchus and truncus intermedius.

**Fig. 2:**
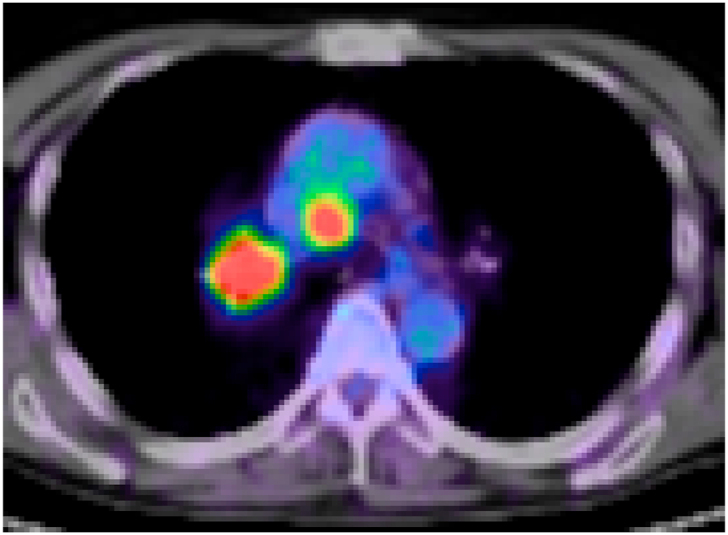
Fused PET/CT image shows FDG concentration in the paratracheal bulky swollen lymph node and primary lung tumor.

A 62-year-old man with lung squamous cell carcinoma (SQ) (cT2aN2M0 Stage IIIA) showing the tumor at the orifice of the right upper lobe was referred (Fig. 1). Pathologic evidence of cancer cell in the paratracheal bulky swollen lymph node was obtained (Fig. 2). As the result of tumor reduction after neoadjuvant chemoradiotherapy including 5 cycles of carboplatin and nab-paclitaxel and thoracic radiation therapy (50 Gy), we performed right upper sleeve lobectomy using ICG imaging as previously described [[2], [3], [4]].

**Fig. 3:**
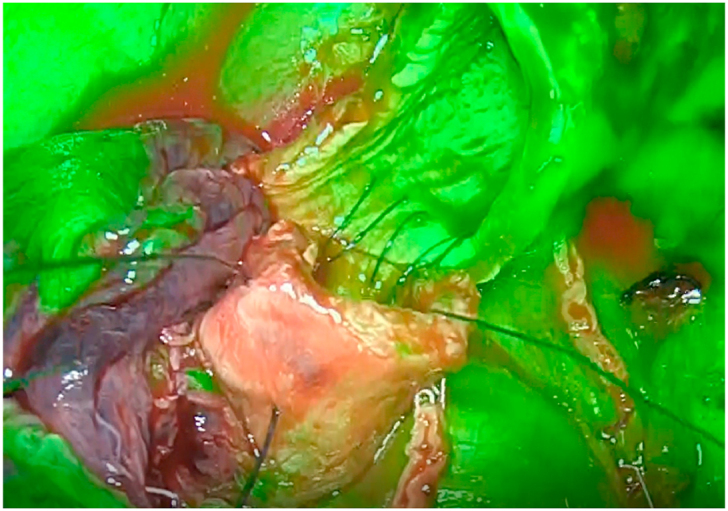
Operative finding showed clear green staining of the upper bronchus, and afterwards, a membranous areas of the truncus intermedius following ICG injection. Supplementary video. Operative findings showed clear green staining of the right main bronchus. Later, a membranous areas of the truncus intermedius was deeplycolored green by near-infrared fluorescence imaging. . (For interpretation of the references to color in this figure legend, the reader is referred to the Web version of this article.)

General anesthesia was performed using a double lumen endotracheal tube for single-lung ventilation. The operation was performed in the lateral decubitus position. This case report was reported in line with the SCARE criteria [5].The right main bronchus and the bronchus intermedius were sharply divided. To clarify the blood supply of the bronchial anastomosis, ICG 5 mg was injected intravenously under fluorescence navigation (1688AIM 4K platform; Stryker, Tokyo, Japan). Operative finding showed clear green staining of the upper bronchus, and afterwards, a membranous area of the truncus intermedius (Fig. 3) (Supplementary video). The blood supply of the bronchial anastomosis judged to be enough. Anastomotic sutures were carried out using a hybrid technique. Bronchoscopy on day 8 after surgery revealed no signs of anastomotic leak or stenosis. The total operation time was 3 hours and 53 minutes. The pathological examination revealed very small number of residual SQ cells without hilar and mediastinal lymph node metastases. Therefore, the lesion was pathologically diagnosed to be at stage IA1 (T1mN0M0). He had a good postoperative course on four months after surgery.

## 3 Discussion

This report describes a first case concerning the blood distribution of the bronchial anastomosis for bronchoplasty using ICG injection under fluorescence navigation. Until now, ICG technique has been explored to create an intersegmental plane for NSCLC [3] or intralobar pulmonary sequestration [4], as we already reported.

The key to the success of the bronchial anastomosis is both tension and the blood flow. However, local tension is inconsistent with blood distribution. Specifically, it may be necessary to release the surrounding tissue to reduce the tension when suturing of the bronchus. However, this maneuver is associated with the risk of reducing the bronchial blood flow [2]. Induction treatment including chemotherapy and radiation has been reported to make cause fibrosis, consequently the decrease of blood supply [6]. Thus, there is a greater risk of anastomotic leakage during bronchoplasty after induction treatment than when performing surgery for local NSCLC. If the blood distribution of the bronchial anastomosis can be made visible after performing the minimum required tension release, the surgeon can be confident of success intraoperatively. Our intraoperative findings clearly showed both the green staining of the upper bronchus and later a membranous area of the truncus intermedius. Thus, ICG imaging can help such a scheduled operation be performed safely by ensuring the blood supply during bronchoplasty.

## Ethics

Written informed consent was obtained from the patient for publication of this case report and accompanying images. A copy of the written consent is available for review by the Editor-in-Chief of this journal on request.

## Sources of funding

There are no sources of funding.

## Author's contribution

HU composed the manuscript, and all the remaining authors provided critical edits to the final draft. All authors read and approved the final manuscript.

## Provenance and peer review

Not commissioned, externally peer reviewed.

## Declaration of competing interest

The authors declare that they have no conflict of interests.
